# Nanoparticle-Based Targeted Drug-Delivery Systems for Cardiomyopathy: Mechanisms and Therapeutic Advances

**DOI:** 10.3390/molecules31173137

**Published:** 2026-09-07

**Authors:** Boshen Liang, Tingwei Zhang, Shidan Tang, Xiaonan Zhou, Sujun Xie, Ziqi Jia, Haopeng Lin, Guangbo Luo, Bin Lin

**Affiliations:** 1 Guangzhou University of Chinese Medicine, Guangzhou 510006, China; 15546459039@163.com (B.L.);; 2 First Clinical Medical College, Guangzhou University of Chinese Medicine, Guangzhou 510400, China

**Keywords:** nanoparticles, cardiomyopathy, active targeting, passive targeting, drug targeting

## Abstract

Cardiomyopathy comprises heterogeneous diseases characterized by structural or functional myocardial abnormalities and caused by conditions such as hypertension and coronary atherosclerotic heart disease. Current management relies mainly on pharmacotherapy and lifestyle interventions, but these approaches are limited by poor bioavailability and adverse effects. Nanoparticles (NPs) can deliver therapeutic drugs to cardiac muscle tissue through passive or active targeting, protecting the drugs they carry from premature degradation. They also show good biocompatibility and minimize damage to healthy tissues. This review summarizes the applications of NPs in cardiomyopathy and their active and passive targeting strategies, and provides a brief overview based on the mechanisms reported in individual published studies. It aims to provide therapeutic strategies for clinical management of cardiomyopathy and a theoretical foundation for future translational research.

## 1. Introduction

Cardiomyopathy is a heterogeneous group of diseases characterized by structural and/or functional abnormalities of the heart muscle. Clinical manifestations often include progressive heart failure, life-threatening arrhythmias, and sudden death, which may ultimately lead to heart failure. Its etiology cannot be entirely attributed to hypertension, coronary atherosclerotic heart disease, valvular heart disease, or congenital heart disease [1]. Cardiomyopathy is primarily classified into five categories based on ventricular morphology and function: dilated cardiomyopathy (DCM), hypertrophic cardiomyopathy (HCM), arrhythmogenic cardiomyopathy (ACM), restrictive cardiomyopathy (RCM), and unclassified cardiomyopathy [2]. According to World Health Organization statistics, approximately 19.8 million people worldwide died from cardiovascular diseases in 2022, accounting for about 32% of all global deaths; 85% of these deaths were attributable to heart attacks and strokes [3,4]. In China, the incidence, prevalence, and age-standardized incidence rate in the general population were 652.21 per 100,000 [3,4]. Cardiomyopathy has become a serious global public health issue, posing a grave threat to health and imposing a substantial economic burden on society. Numerous clinical approaches are available to reduce cardiovascular morbidity and mortality in high-risk individuals or patients with confirmed diagnosis, including lifestyle interventions, pharmacological therapy (blood glucose control, lipid management, myocardial protection, and heart failure treatment), and surgical interventions [5,6]. However, after 2 months of continuous treatment with 1 mg/kg of the loop diuretic furosemide, TGF-β- and MAPK-related inflammatory and profibrotic pathways were activated, thereby promoting extracellular matrix remodeling and myocardial fibrosis [5]; beta-blockers have significant negative inotropic effects and may worsen heart failure and bradycardia; and digitalis drugs have a very narrow therapeutic window and provide short-term symptom relief [6,7,8]. Furthermore, gene therapy offers a promising alternative to traditional therapies; however, the systemic delivery of nucleic acids is limited by enzymatic degradation and poor tissue targeting [9].

Nanoparticles (NPs) are widely used as carriers for intracellular drug delivery. Appropriate nanocarriers can enhance blood–brain barrier penetration, targeting, encapsulation efficiency, drug-loading capacity, half-life, bioavailability, and safety. Since the early 21st century, nanotechnology has become a major research field [10]. NPs are classified as organic or inorganic. Organic NPs improve delivery efficiency, are easy to synthesize and modify, and possess good physicochemical properties and biocompatibility. Inorganic NPs offer high controllability, minimal side reactions, high bioavailability, large surface area, and excellent drug release control capabilities. Organic NPs include liposomes, micelles, vesicles, polylactic-co-glycolic acid nanoparticles (PLGA), hydrogels, and dendrimers; inorganic nanomaterials include elemental metal, metal oxide, and carbon NPs [11]. In addition, NPs can deliver drugs directly to target tissues and cells, offering potential for optimizing the treatment of various cardiomyopathies. Accordingly, this article reviews recent advances in nanodelivery strategies, focusing on inorganic and organic NPs, aiming to propose new approaches for the clinical treatment of various cardiomyopathies. Furthermore, it discusses genetic and viral nanocarriers as future directions.

## 2. Pathogenesis of Cardiomyopathies

### 2.1. Mitochondrial Damage

#### 2.1.1. Impaired Mitochondrial Structure

Mitochondria are enclosed structures composed of two superimposed membranes, are typically rod- or granule-shaped, and are primarily divided into the outer membrane, intermembrane space, inner membrane, and matrix. Mitochondria are semi-autonomous organelles that possess their own genetic material, mtDNA. mtDNA mutations can impair oxidative phosphorylation (OXPHOS) and respiratory chain function in cardiomyocytes, leading to reduced mitochondrial ATP production and increased ROS generation, which ultimately contributes to chronic heart failure. Studies have shown that mtDNA mutations are present in the cardiomyocytes of approximately one-quarter of DCM patients with mitochondrial abnormalities, and mtDNA point mutations are also found in the peripheral blood of children with primary DCM [12,13].

Mitochondrial DNA mutations associated with DCM should be classified into germline and somatic variants. Germline mitochondrial DNA variants are inherited maternally, and their level of heterogeneity may vary across different tissues and generations; therefore, their clinical manifestations depend in part on whether the mutational burden in the myocardium exceeds a functional threshold. In contrast, somatic mitochondrial DNA variants arise after fertilization and may accumulate in the myocardium during the aging process or as a result of oxidative and metabolic stress. These variants are not heritable and may be confined to specific tissues; therefore, peripheral blood testing may underestimate their abundance in the myocardium [14]. This distinction affects treatment options: germline mutations can be addressed through genetic counseling, prevention of maternal transmission, or strategies to modulate heterogeneity, whereas somatic mutations require cardiac-specific detection and targeted mitochondrial interventions. Germline mtDNA variants are transmitted maternally and may cause familial DCM by disrupting oxidative phosphorylation and myocardial energy production; somatic mtDNA variants may exacerbate mitochondrial dysfunction, leading to sporadic or late-onset DCM [15].

FUN14 domain-containing protein 1 (FUNDC1) is a mitochondrial membrane protein that plays a crucial regulatory role in maintaining mitochondrial integrity. Studies have confirmed that FUNDC1 is downregulated in both DCM and diabetic cardiomyopathy [16,17].

#### 2.1.2. Impaired Mitochondrial Function

Mitochondria are the primary site of oxidative phosphorylation and adenosine triphosphate (ATP) synthesis and serve as the center of energy metabolism in the body. Calcium dysregulation is a major contributor to mitochondrial dysfunction. Mitochondria, together with the endoplasmic reticulum and extracellular matrix, regulate Ca^2+^ uptake and release to maintain intracellular calcium homeostasis. Calcium/calmodulin-dependent protein kinase II (CaMKII) is a key regulator of calcium homeostasis in cardiomyocytes. However, the mechanisms and consequences of calcium (Ca^2+^) dysregulation differ across various types of cardiomyopathy. In DCM, overexpression of cardiac mitochondrial CaMKII triggers adverse metabolic reprogramming, impairs ATP production, and leads to ventricular dilation and systolic dysfunction [18]. In contrast, in a pediatric HCM model carrying the TPM1-p.E181K mutation, inhibition of the CaMKII/HDAC4 signaling pathway disrupts the organizational structure of the myofibrils and increases myocardial contractility, thereby reducing ventricular compliance and impairing diastolic filling [19]. In arrhythmogenic cardiomyopathy (ACM), abnormalities in calcium handling within cardiac stromal cells have been identified; these abnormalities tend to promote fibro-adipogenic remodeling rather than directly causing mitochondrial energy depletion [20]. In diabetic cardiomyopathy, activation of the TLR4/CaMKII/NLRP3 pathway links calcium-dependent signaling to inflammatory responses and cardiomyocyte pyroptosis [21]. Therefore, although calcium dysregulation is present in all these forms of cardiomyopathy, the affected cell types, upstream triggers, downstream signaling pathways, and functional consequences depend on the specific subtype.

Autophagy transports abnormal cellular components, including misfolded proteins, damaged organelles, and accumulated harmful substances, to lysosomes for degradation and recycling. It supports cellular survival and functions as a self-protective mechanism [22]. Cardiovascular diseases, particularly cardiomyopathies, are closely associated with reduced autophagic flux. Because cardiomyocytes depend on mitochondrial health, mitophagy—a form of selective autophagy of damaged mitochondria—is particularly important. Disrupted autophagy–mitophagy balance promotes adverse cardiac remodeling and the progression of cardiomyopathy by accelerating mitochondrial dysfunction, oxidative damage, and cardiomyocyte death. The initiation of autophagy is synergistically regulated by AMP-activated protein kinase (AMPK) and the mammalian target of rapamycin complex 1 (mTORC1), which play complementary roles in cellular energy and nutrient homeostasis. Under stress conditions, AMPK promotes autophagy by directly activating ULK1 and inhibiting mTORC1; conversely, under nutrient-rich conditions, activated mTORC1 inhibits ULK1. Once this inhibitory regulation is lifted, ULK1 can form a complex with Atg13 and FIP200 and initiate autophagosome formation [23,24]. Subsequently, activated ULK1 acts in concert with Beclin1 to promote the formation of the Atg14–Vps15–Vps34 phosphoinositide 3-kinase complex, thereby promoting autophagy [25]. Consistent with this regulatory framework, metformin activated AMPK-dependent autophagy and inhibited the NLRP3 inflammasome in a mouse model of diabetic cardiomyopathy, thereby reducing myocardial inflammation and injury [26].

### 2.2. Myocardial Inflammation Injury

Inflammation is a protective response to tissue damage and a dynamic process involving injury, defense, and repair. Its fundamental pathological changes include tissue degeneration, exudation, and proliferation. Autophagy can suppress inflammatory responses, potentially by directly inhibiting inflammasomes and indirectly clearing inflammatory stimuli, such as damaged organelles or pathogenic microorganisms, thereby protecting cells from excessive and prolonged inflammation [27].

The NLRP3 inflammasome consists of NLRP3, apoptosis-related speck-like protein (ASC), and caspase-1. It is widely expressed in cardiomyocytes, endothelial cells, macrophages, and fibroblasts and plays a significant role in the onset and progression of cardiomyopathy [28]. Characterization of doxorubicin-induced DCM has demonstrated that ROS-dependent activation of the NLRP3 inflammasome and pyroptosis links NLRP3 to the pathogenesis of DCM; NLRP3 inhibitors may reverse DCM-induced myocardial damage [29]. The pathological significance of NLRP3 inflammasome components depends on their functional location and the specific subtype of cardiomyopathy. Caspase-1 cleaves GSDMD and promotes the maturation of IL-1β and IL-18; meanwhile, the N-terminal fragment of GSDMD forms membrane pores, leading to cell membrane rupture and the release of inflammatory mediators. Thus, caspase-1 and GSDMD influence the pyroptosis process, while IL-1β and IL-18 primarily exacerbate the inflammatory response and lead to cardiac remodeling [30]. In diabetic cardiomyopathy, metabolic stress, calcium homeostasis disruption, mitochondrial reactive oxygen species (ROS), and damaged mitochondrial DNA activate NLRP3, which, in turn, triggers caspase-1 activation and the release of IL-1β and IL-18. Caspase-1 and GSDMD play direct roles in the pyroptosis process of cardiomyocytes and are key factors in this pathway. IL-1β also plays an important role by promoting immune cell recruitment, fibroblast activation, extracellular matrix deposition, and chronic myocardial inflammation, whereas the independent role of IL-18 has been relatively less studied [31]. A similar pattern is observed in non-ischemic and doxorubicin-induced DCM, where the caspase-1–GSDMD pathway is associated with cardiomyocyte loss, ventricular dilation, fibrosis, and ejection fraction impairment. In HCM, elevated expression levels of caspase-1, GSDMD, IL-1β, and IL-18 are associated with activation of the NLRP3 pathway. In ACM, IL-1β may play a more critical role: studies of human myocardial tissue and Dsg2-mutant mice have revealed the presence of NLRP3-expressing macrophages and elevated IL-1β levels within inflammatory-fibrotic foci, while IL-1β neutralization reduces fibrosis, preserves cardiac function, and alleviates electrophysiological abnormalities [32].

The cyclic guanosine monophosphate-adenosine monophosphate (cGAMP) synthase (cGAS)-stimulator of interferon genes (STING) signaling pathway is an intracellular immune response mechanism activated by cytoplasmic DNA and typically plays a crucial role in tumor immunity [33]. This pathway can also improve cardiac function and reduce cardiomyocyte hypertrophy in HCM by inhibiting CGAS/STING/NLRP3 [34]. New evidence suggests that the cGAS–STING signaling pathway may be involved in a molecularly defined subgroup of sporadic HCM, rather than representing a universal mechanism for classic myotubular HCM. A somatic NAP1L1 p.D349E variant identified in this study in patients with sporadic HCM—a condition in which genetic testing is often challenging—was shown to disrupt nucleosome formation, promote the release of nuclear DNA into the cytoplasm, and activate the cGAS–STING–IFN signaling pathway [35]. NLRP3 selectively interacts with ASC to form the NLRP3-ASC complex. This complex may act as an upstream activator of the NF-κB signaling pathway, participating in the regulation of NF-κB activity. Studies have demonstrated that the TLR4/ZNF480/AP-1/NF-κB signaling pathway is associated with myocardial injury [36].

### 2.3. Oxidative Stress

Oxidative stress results from an imbalance between oxidants and antioxidants, leading to excess reactive oxygen species (ROS) that damage lipids, proteins, and DNA. Although oxidative stress is inevitable in oxygen-rich environments, moderate oxidative stress helps maintain normal cellular function by regulating redox-sensitive signaling pathways, such as NF-κB and Nrf2/Keap1. Oxidative stress responses increase inflammatory cytokines, including TNF-α and IL-1β, which contribute to inflammation [37].

In patients with DCM, the Nrf2/Keap1 ratio is significantly elevated, whereas the expression of antioxidants such as NQO1, SOD1, and GPX3 is markedly reduced [38]. The PI3K/AKT signaling pathway is markedly upregulated in animal models of HCM, while Nrf2 expression is downregulated. Furthermore, HCM development is associated with apoptosis, as evidenced by decreased expression of Bcl-2 and Bcl-xL and increased caspase-3 activity [39].

### 2.4. Endoplasmic Reticulum Stress

Endoplasmic reticulum (ER) stress is triggered by the accumulation of misfolded proteins and is resolved by the activation of the unfolded protein response (UPR). However, prolonged stress can lead to apoptosis. ROS induce ER stress by oxidizing proteins and disrupting their folding state, while ER stress, in turn, can increase ROS production through mechanisms such as Endoplasmic Reticulum Oxidoreductase 1 (ERO1), thereby exacerbating the oxidative stress response and creating a vicious cycle [40].

The production of mtROS activates the Keap1–Nrf2 pathway, inducing the expression of antioxidant genes such as HO-1, NQO1, SOD, and GPX. When ROS production exceeds this protective capacity, sustained oxidative stress damages mitochondrial components and disrupts protein folding in the endoplasmic reticulum. The resulting endoplasmic reticulum stress activates the unfolded protein response; if this condition persists for too long, it will further increase ROS production, disrupt calcium homeostasis, and exacerbate mitochondrial dysfunction. Subsequently, excessive ROS and damaged mtDNA activate inflammatory signaling pathways such as NF-κB and NLRP3, thereby promoting inflammatory responses and cardiomyocyte death, leading to fibroblast activation, extracellular matrix deposition, and adverse myocardial remodeling [41].

### 2.5. Interactions Among Pathogenic Mechanisms

Mechanisms such as mitochondrial dysfunction, oxidative stress, and inflammation can influence one another. When the mitochondrial membrane is damaged, mitochondria produce excessive amounts of mitochondrial reactive oxygen species (mtROS), leading to oxidative stress. Excessive mtROS, in turn, oxidizes proteins, lipids, and DNA within the mitochondria, further impairing oxidative phosphorylation and ATP production [42]. mtROS, potassium efflux, and oxidized mitochondrial DNA (mtDNA) can activate the NLRP3 inflammasome, which, in turn, activates caspase-1, leading to elevated levels of IL-1β and IL-18 and potentially triggering pyroptosis. At the same time, increased mitochondrial membrane permeability and an insufficient capacity to clear damaged mitochondria via mitochondrial autophagy result in the accumulation of mtDNA in the cytoplasm. mtDNA in the cytoplasm is recognized by cyclic GMP-AMP synthase (cGAS), which generates cyclic GMP-AMP and activates stimulator of interferon genes (STING). The subsequent activation of the TBK1–IRF3 and NF-κB pathways induces type I interferons and other pro-inflammatory mediators, exacerbating the inflammatory response [43,44].

**Figure 1:**
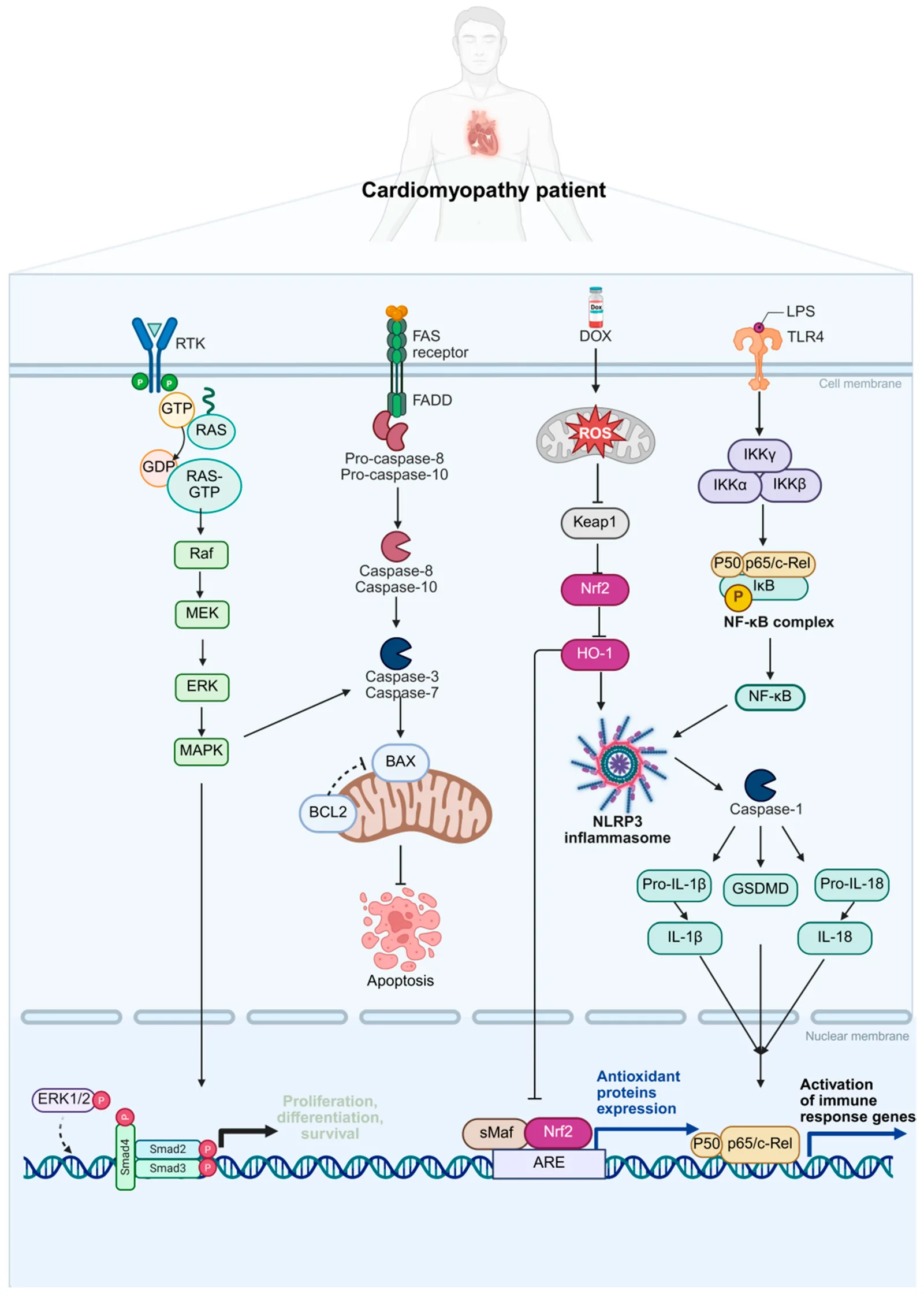
Possible functional impairments and mechanisms involved in the pathogenesis of various cardiomyopathies. When the body is exposed to external factors such as infection or poisoning, an inflammatory response is triggered. This activates NLRP3, which, in turn, leads to apoptosis and elevated levels of inflammatory mediators such as interleukins in the body. At the same time, the body’s antioxidant system becomes unbalanced, and the accumulation of reactive oxygen species causes oxidative stress that disrupts cellular function, induces endoplasmic reticulum stress, and leads to an imbalance in calcium ion homeostasis, resulting in mitochondrial dysfunction. RTK, receptor tyrosine kinase; GTP, guanosine triphosphate; GDP, guanosine diphosphate; RAS, rat sarcoma viral oncogene homolog; RAS- GTP, ras-related gtp-binding proteins; RAF, rapidly accelerated fibrosarcoma; MEK, mitogen-activated protein kinase kinase; ERK, extracellular signal-regulated kinase; MAPK, mitogen-activated protein kinase; ERK1/2, extracellular signal-regulated kinase 1/2; Smad2,sma- and mad-related protein 2; Smad3, sma- and mad-related protein 3; Smad4, sma- and mad-related protein 4; FAS, fas cell surface death receptor; FADD, fas-associated death domain protein; BAX, bcl2 associated x protein; BCL2, b-cell lymphoma 2;DOX, doxorubicin; ROS, reactive oxygen species; Keap1, kelch-like eCH-associated protein 1; Nrf2, nuclear factor erythroid 2-related factor 2; HO-1, heme oxygenase-1; NLRP3 inflammasome, nlr family pyrin domain containing 3 inflammasome; sMaf, small maf proteins; ARE, antioxidant response element; LPS, lipopolysaccharide; TLR4, Toll-like receptor 4; IKKy, inhibitor of nuclear factor kappa b kinase subunit gamma; IKKα, inhibitor of nuclear factor kappa b kinase subunit alpha; IKKβ, inhibitor of nuclear factor kappa b kinase subunit beta; P50, nuclear factor kappa b subunit 1; p65, rela proto-oncogene, nf-kb subunit; c-rel, rel proto-oncogene transcription factor; IkB, inhibitor of nuclear factor kappa B; P, phosphate group; NF-KB complex, nuclear factor kappa B transcription factor complex; NF-KB, nuclear factor kappa B; Pro-IL-1β, pro–interleukin-1 beta; GSDMD, gasdermin d; Pro-IL-18, pro–interleukin-18; IL-1β, interleukin-1 beta; IL-18, interleukin-18; Caspase-1, cysteine-aspartate protease 1; Caspase-3, cysteine-aspartate protease 3; Caspase-7, cysteine-aspartate protease 7; Caspase-8, cysteine-aspartate protease 8; Caspase-10, cysteine-aspartate protease 10.

The interactions among these signaling pathways play a significant role in the onset and progression of cardiomyopathy. Their concurrent activation disrupts myocardial energy metabolism and calcium homeostasis, reduces myocardial contractility, and promotes apoptosis, pyroptosis, and other forms of cell death [45]. Furthermore, persistent oxidative and inflammatory damage activates cardiac fibroblasts, enhancing extracellular matrix deposition, which leads to myocardial fibrosis, ventricular hypertrophy or dilation, electrical instability, and progressive cardiac dysfunction. Although the relative contributions of these pathways vary across different cardiomyopathy subtypes and disease stages, their interactions represent a common mechanism linking cellular stress to adverse myocardial remodeling and heart failure [46]. We have summarized the pathogenesis of cardiomyopathy in Figure 1.

## 3. Properties of Nanoparticles

The combination of nanotechnology and biomedicine holds great promise. With the rapid advancement of nanotechnology, drug-delivery systems based on nanocarriers have attracted widespread attention. NPs possess superior properties not found in many traditional materials, such as increasing the systemic circulation time of drugs, reducing off-target cytotoxicity, improving drug solubility, lowering the required dosage, combining diagnostic and therapeutic agents to form therapeutic drugs, and enhancing drug accumulation at specific sites. They hold great promise for the treatment of various types of cardiomyopathy [47]. Nanoparticle platforms can be broadly categorized into: organic nanocarriers (including liposomes, micelles, dendrimers, and polymer nanoparticles); inorganic nanomaterials (including gold, silver, silica, iron oxide, and carbon-based nanoparticles); and bio-derived systems (including exosomes and other extracellular vesicles) [48,49,50]. The efficiency of their delivery is influenced by factors such as size, shape, surface characteristics, hydrophobic nature, and both biophysical and chemical attributes. These NPs can be designed to specifically target affected regions in the body and facilitate controlled release of the drug, resulting in enhanced bioavailability, lower dosage requirements, shorter treatment times, and reduced side effects [51].

Cancer treatment has long been one of the earliest, most extensively studied, and clinically most mature branches of this field. Passive targeting of NPs can leverage the pathological characteristics of tumor vasculature to promote their accumulation within tumors; active targeting, on the other hand, utilizes ligands and other mechanisms to facilitate receptor-mediated recognition and endocytosis by cancer cells. NPs can induce mitochondrial apoptosis or trigger ferroptosis through mechanisms such as oxidative stress. These principles can serve as a foundation for designing targeted nanodelivery systems for cardiomyopathy [52].

Inorganic nanomaterials have become a highly promising therapeutic platform in the biomedical field. They can regulate redox homeostasis. For example, certain inorganic nanoparticles exhibit enzyme-mimicking or catalytic activity, enabling them to scavenge ROS and alleviate oxidative damage; conversely, certain metal and metal oxide nanoparticles can promote the generation of reactive oxygen species through mechanisms such as surface reactions and the release of metal ions. These effects depend on factors such as their composition, oxidation state, particle size, surface defects, coating, dosage, and biological environment [53]. Additionally, their extensive specific surface area and abundance of active sites on the surface allow for effective multi-enzyme cascade catalysis, thereby greatly improving catalytic efficiency. Metal-organic framework-based nanomaterials have further expanded these application areas, as their high porosity, tunable composition, and easily functionalizable surfaces enable the loading and controlled release of small molecules, biomacromolecules, and diagnostic reagents [54]. Simultaneously, these NPs can prevent protein denaturation or proteolytic breakdown, which assists in overcoming biological obstacles like the blood–brain barrier and intestinal barrier, thereby enhancing drug delivery and reducing drug degradation [55]. However, the clinical application of inorganic nanoparticles is limited by poor biodegradability, prolonged organ accumulation, ion release, oxidative stress, inflammation, and potential long-term toxicity [56]. We have summarized the relevant information in Table 1.

**Figure 2:**
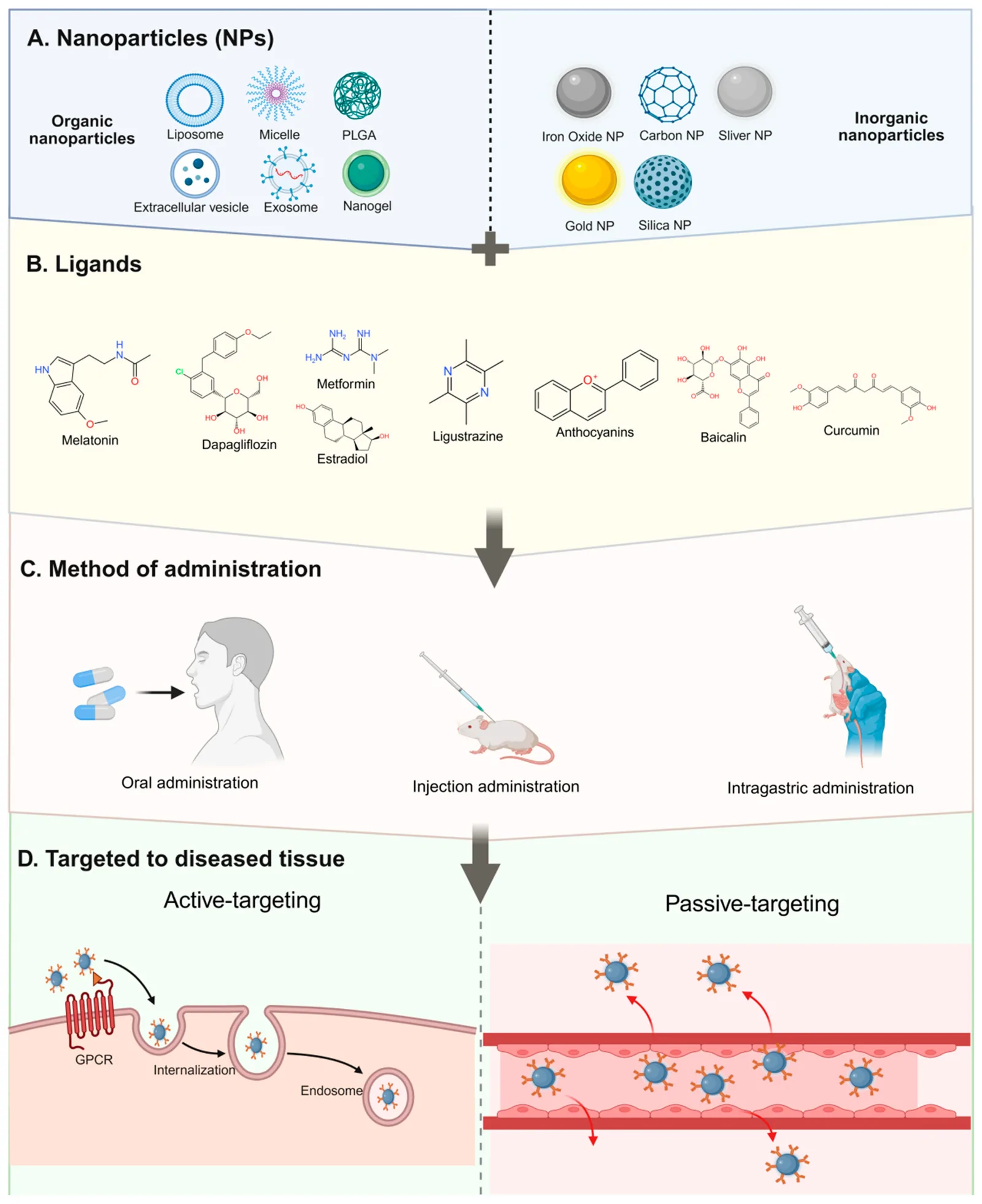
Classification and Delivery of Nanoparticles. (**A**) Classification of organic and inorganic nanoparticles. (**B**) Major ligands carried by nanoparticles. (**C**) Nanoparticles can be delivered to target sites via various routes of administration, including oral, gastric, or injection. (**D**) Nanoparticles are transported to damaged sites via active or passive transport. PLGA, poly(lactic-co-glycolic acid); GPCR, g protein–coupled receptor.

Furthermore, NPs can serve purposes such as radiolabeling or functional modification, which enhances their significance in targeted drug delivery, cellular imaging, and molecular detection. These characteristics support NPs applications in medicine. Figure 2 illustrates the classification of common nanoparticles, ligand information, and the targeted delivery process.

## 4. Nanoparticle Therapy for Cardiomyopathy

Organic nanoparticles include liposomes, PLGA, chitosan, micelles, hydrogels, and others, and are highly suitable for biomedical applications. Inorganic nanocarriers, which consist of metals, metal oxide nanoparticles, and semiconductor materials, show great potential for improving the solubility and bioavailability of poorly soluble drugs.

### 4.1. Nanoparticle Therapy for DCM

#### 4.1.1. PLGA Therapy for DCM

PLGA and its modified nanosystems can contribute to the treatment of DCM through various mechanisms, including myocardial repair, reduction in drug-induced cardiotoxicity, inhibition of myocardial remodeling. Cardiac homing peptide-modified, miR-30b-5p-loaded PEG-PLGA NPs alleviate myocardial fibrosis, inflammation, and ventricular remodeling by targeting TGFBR2 and inhibiting TGF-β/TGFBR2-related signaling pathways [57]. In future precision therapy and gene therapy delivery, Switala et al. found that cargo-less PLGA particles, known as enhancer polymers, are taken up primarily by Kupffer cells in the liver rather than deposited directly in the heart but can significantly improve cardiac delivery efficiency of AAV vectors and virus-like NPs while reducing off-target accumulation in the liver. Given that hereditary DCM and some cases of idiopathic DCM are closely associated with genetic mutations, this study suggests that PLGA can serve not only as a carrier for traditional drugs or nucleic acids but also as a cardiac-targeted delivery enhancer, offering a new direction in nanomedicine for gene delivery, gene editing, and nucleic acid drug therapy in DCM [58]. To address chemotherapy-induced secondary DCM, Drinkovic et al. prepared PLGADOX and compared it with ConvDOX and LipoDOX. The results showed that ConvDOX caused significant myocardial fiber necrosis and inflammatory responses, whereas PLGADOX induced milder myocardial damage, primarily reflected by limited effects on mitochondrial metabolism. This indicates that PLGA-encapsulated DOX can mitigate the cardiotoxicity of anthracycline drugs, providing a nanomedicine strategy for preventing chemotherapy-associated DCM [59].

Overall, PLGA-based nanoparticles contribute to DCM treatment by reducing drug-induced cardiotoxicity, delivering anti-fibrotic and anti-remodeling nucleic acids, and enhancing cardiac-targeted drug and gene delivery.

#### 4.1.2. Exosome Therapy for DCM

Exosomes are natural nanoscale membrane-bound extracellular vesicles (EVs). They have excellent biocompatibility, low immunogenicity, and tissue- or cell-targeting properties, and they mediate intercellular communication and functional regulation. Because they deliver cargo to recipient cells and influence diverse biological processes, they are promising natural nanoscale platforms for disease diagnosis, biomarker discovery, and innovative drug development [60].

Exosomes perform crucial roles in the pathogenesis, diagnosis, and therapeutic intervention of DCM, particularly at the genetic level. circRNAs carried by circulating exosomes in DCM patients act as information carriers and participate in myocardial remodeling, inflammation, oxidative stress, apoptosis, and fibrosis. Xu et al. found that plasma exosomes from patients with DCM complicated by heart failure have a specific circRNA profile, including 49 differentially expressed circRNAs, suggesting that exosomal circRNAs may serve as potential biomarkers for DCM [61]. Furthermore, the same study identified 92 differentially expressed miRNAs in plasma exosomes from patients with DCM complicated by heart failure, indicating that plasma exosomal miRNAs may serve as stable nanoscale biomarkers and potential targets for exosome-based diagnostic and therapeutic strategies [62]. Exosomes also serve as mediators of intercellular communication and may contribute to myocardial remodeling, fibrosis, and hypertrophy. Jiang et al. found that serum exosomes from pediatric DCM patients are taken up by NRVMs and iPSC-CMs, inducing ANP and BNP expression and cardiomyocyte hypertrophy, suggesting their value in early diagnosis of pediatric DCM [63]. Gong et al. showed that these exosomes inhibit autophagy via the Akt/mTOR pathway, thereby increasing Col1A1, ANP, and BNP expression and exacerbating DCM-associated myocardial fibrosis and hypertrophy [64]. DOX-induced chemotherapy-associated cardiomyopathy can also be improved through multiple mechanisms—including anti-inflammatory, anti-apoptotic, and anti-fibrotic effects, as well as the regulation of cardiac remodeling—mediated by exosomes and their associated nanodelivery systems. Therapeutic exosomes from various sources, such as those derived from mesenchymal stem cells, trophoblast stem cells, and cardiac stem cells are effectively taken up by cardiomyocytes or cardiac tissue. In animal models, they increase left ventricular ejection fraction (LVEF) and reduce apoptosis, inflammation, fibrosis, and ventricular remodeling. MSC-Exos promote macrophage polarization from the pro-inflammatory M1/Ly6C^high^ phenotype to the anti-inflammatory M2/Ly6C^low^ phenotype by activating the JAK2/STAT6 pathway, thereby reducing IL-1, IL-6, TNF-α, and Bax expression and alleviating myocardial inflammation. TSC-Exos deliver let-7i-5p to inhibit YAP/Hippo signaling, downregulating Cleaved-Caspase3, CTGF, and ANP and reducing apoptosis, inflammation, and fibrosis. CSC-Exos also improve cardiac dysfunction in DOX-induced chemotherapy-associated cardiomyopathy and reduce myocardial cell apoptosis and fibrosis [65,66,67]. In addition, the heart-targeted liposome Lipo@miR-185-5p inhibitor combines exosome-derived miRNA regulation with nanodelivery to modulate BCL-2, BAX, Cleaved-Caspase3, dihydrolipoamide S-acetyltransferase, dihydrolipoamide S-succinyltransferase, and ferredoxin 1, thereby inhibiting apoptosis, oxidative stress, and DNA damage and improving cardiac function in mice with DOX-induced chemotherapy-associated cardiomyopathy [68]. Exosomes derived from different cell sources have opposing effects on myocardial fibrosis and function, primarily due to differences in the origin and state of the donor cells. Exosomes derived from healthy stem cells or progenitor cells typically carry miRNAs and proteins with cardioprotective effects, which can inhibit inflammation, apoptosis, and fibrosis. However, exosomes released by stressed or diseased cardiomyocytes may contain pathological signals that activate cardiac fibroblasts and lead to cardiac remodeling. Their biological effects are also influenced by the type of recipient cells, the stage of the disease, dosage, and tissue distribution.

Consistent with this source-dependent heterogeneity, exosomes released from diseased cardiomyocytes can also exert harmful effects on the myocardium. Exosomes derived from cardiomyocytes in DCM can be taken up by cardiac fibroblasts, where they promote myocardial fibrosis and impair cardiac function by enriching miR-218-5p, inhibiting TNFAIP3, activating the TGF-β/Smad2 pathway, and upregulating fibrosis markers such as Col1a, Col3a, and CTGF [69].

#### 4.1.3. EVs Therapy for DCM

EVs are nanoscale membrane-bound vesicles actively released into the extracellular environment. They mediate intercellular communication, immune regulation, inflammatory responses, tissue repair, and disease progression. Because they reflect the physiological or pathological state of their source cells and transfer molecular information they carry to recipient cells, EVs serve as liquid biopsy biomarkers for disease diagnosis and prognosis assessment and natural nanodelivery systems for drugs, nucleic acids, and proteins. They, therefore, hold substantial value for precision diagnosis and treatment of cardiovascular and other diseases [70].

EVs can participate in the pathological progression, molecular diagnosis, and therapeutic intervention of DCM by carrying active molecules such as mRNA and noncoding RNA. Circulating EVs from DCM patients exhibit distinct disease-specific molecular characteristics. Roura et al. isolated plasma-derived EVs via SEC and combined this with LC-MS/MS proteomic analysis, revealing a unique protein profile in DCM-EVs. This profile showed significant enrichment of fibrinogen α/β/γ chains, serotransferrin, α-1-antitrypsin, clusterin, and various other proteins. These proteins are primarily associated with stress responses, platelet degranulation, coagulation, and complement-related pathways, suggesting that circulating EVs may serve as an important source for liquid biopsy and disease progression monitoring in DCM [71]. EVs derived from different sources or generated by various induction methods can improve DCM through immunomodulatory, anti-inflammatory, and anti-fibrotic mechanisms. In a mouse model of DOX-induced cardiomyopathy, endothelial cell-derived exosomes overexpressing KLF2 (KLF2-EVs) improved LVEF and LVFS. This model represents chemotherapy-induced secondary DCM. KLF2-EVs alleviate cardiac inflammation by inhibiting CCR2-dependent monocyte migration, suggesting their potential for treating anthracycline-induced cardiac injury [72]. Sun et al. demonstrated that low-intensity pulsed ultrasound (LIPUS) can induce endothelial cells to release miR-99a-rich EVs. After uptake by CD4+ T cells, these EVs suppress mTOR and TRIB2 expression, reduce glycolysis, and shift the Th17/Treg immune balance toward an anti-inflammatory state, thereby alleviating myocardial hypertrophy, inflammation, and fibrosis in inflammatory DCM. The findings indicate that LIPUS, by activating endothelial cell EV-miR-99a-mediated immune remodeling, serves as a noninvasive nanovesicle-induced therapeutic strategy for DCM and offers a promising EV-based nanomedicine approach [63]. In a case report on heart failure, Menasché et al. were the first to apply an EV-enriched secretome derived from human iPSC-derived cardiovascular progenitor cells to patients with severe nonischemic DCM-related heart failure. Repeated intravenous infusions were well tolerated, with no serious adverse events, ventricular arrhythmias, or significant immune reactions. After 6 months, NT-proBNP levels decreased, LVEF improved, and LVEDV/LVESV decreased. This study suggests that a CPC-derived EV-enriched secretome can serve as a safe nanomedicine therapeutic strategy for DCM-related heart failure [73]. In DCM, EVs serve both as stable nanoscale liquid biopsy biomarkers reflecting molecular changes and inflammatory/coagulation states and as natural therapeutic nanovesicles that deliver miRNAs, proteins, and immunomodulatory signals. They therefore provide a crucial research foundation for precise diagnosis, disease progression monitoring, and cell-free nanomedicine treatment of DCM.

#### 4.1.4. Carbon Nanoparticle Therapy for DCM

Among inorganic nanomaterials, carbon nanomaterials (CNPs) have potential for addressing DCM-associated electrophysiological abnormalities, impaired contractility, myocardial repair, and disease monitoring owing to their electrical conductivity, mechanical strength, flexibility, and biocompatibility. Because DCM often involves ventricular dilation, reduced contractility, and abnormal electromechanical coupling; therefore, carbon nanomaterials with conductive and mechanical support capabilities can provide a new material foundation for its treatment.

At the cellular level, multi-walled carbon nanotubes (MWCNTs) enhance electrical activity and contractility in neonatal rat ventricular myocytes. MWCNTs promote Cx43, plakoglobin, desmoplakin, and N-cadherin expression and increase field potential frequency, calcium transient frequency and amplitude, and cardiomyocyte shortening. They also downregulate miR-135a-5p, relieving its inhibition of the sodium/calcium exchanger NCX1 and enhancing calcium handling, contraction-related gene expression, and oxidative phosphorylation activity [74].

#### 4.1.5. Gold Nanoparticle Therapy for DCM

Among inorganic nanomaterials, gold nanoparticles (AuNPs) have demonstrated substantial potential in biomedicine, material science, and bioanalytical chemistry due to their surface modifiability, near-infrared absorption, photothermal conversion capacity, and drug-delivery potential. AuNPs are biocompatible, can reduce immune rejection risk, and penetrate biological barriers such as the blood–brain barrier. Their large surface area enables loading of drugs, proteins, or genetic material through covalent or noncovalent binding. Their surface plasmon resonance and light-scattering make them suitable for phototherapy and photodiagnostics [75,76]. AuNPs have research value in targeted drug delivery, therapeutic monitoring, and myocardial protection in heart failure associated with cardiomyopathy. Higuchi et al. used PEG-modified gold nanorods (GNRs) to examine distribution in a mouse model of inflammatory cardiomyopathy with cardiac-specific TNF-α overexpression, a model characterized by myocardial inflammation, ventricular dilation, and cardiac dysfunction. PEG-modified GNR levels were significantly higher in transgenic failing hearts than in wild-type hearts, and myocardial gold content correlated positively with ventricular weight/body weight. These findings suggest that GNRs accumulate in diseased hearts as myocardial inflammation and ventricular hypertrophy increase, supporting targeted imaging and drug delivery in DCM-related inflammatory remodeling [77]. Further studies confirmed the cardioprotective effects of 30 nm AuNPs and verapamil conjugates, showing improved myocardial contractility, reduced pleural effusion, and lower mortality in DCM rats, with greater efficacy compared to levosimendan alone. Furthermore, ultrasonic cavitation technology further enhanced AuNP delivery to cardiomyocytes and mitochondria, supporting targeted drug-delivery systems for DCM [78].

However, the use of gold nanomaterials in DCM also requires careful consideration of safety limits. At the same time, the safety risks associated with non-medical colloidal metal supplements should be distinguished from those associated with therapeutic AuNPs. Archer reported a case of a patient who developed DCM and left bundle branch block after consuming one teaspoon of a 20 ppm colloidal gold preparation daily for approximately three consecutive months and intermittently taking colloidal silver for up to seven years. Therefore, long-term use of inorganic nanoparticle preparations should be avoided in clinical practice [79].

#### 4.1.6. Silver Nanoparticle Therapy for DCM

Silver nanoparticles (AgNPs) possess antibacterial, anti-inflammatory, biosensing, and cell differentiation-regulating properties and show potential in cardiovascular tissue engineering and regenerative medicine.

AgNPs exhibit biological effects that are concentration- and context-dependent. In H9c2 cardiac progenitor cells, cell viability decreased in a dose-dependent manner following 48 h exposure to AgNPs at concentrations ranging from 0 to 10 µg/mL. At the IC_50_ concentration for a specific formulation, AgNPs increased ROS production, reduced mitochondrial ATP levels, and upregulated the expression of AMPK1α, Atg5, Beclin1, and LC3BII, indicating ROS-mediated mitochondrial dysfunction, autophagy, and apoptosis [80]. AgNPs also reduced mitochondrial membrane potential, the NAD+/NADH ratio, and ATP levels through AMPK/mTOR-related autophagy dysfunction, whereas sodium selenite partially attenuated these toxic effects [81].

Adibkia et al. found that 2.5 µg/mL AgNPs promote cardiomyogenic differentiation of BM-MSCs, extend telomere length, and upregulate the expression of TERT, cyclin D, and Wnt3/β-catenin-related genes, suggesting that AgNPs may enhance the differentiation of BM-MSCs into cardiomyocytes through telomere length extension and the Wnt3/β-catenin signaling pathway [82]. The relationship between AgNPs and DCM is primarily manifested as follows: under high-risk exposure, AgNPs may exacerbate DCM-related myocardial damage through oxidative stress, mitochondrial damage, and abnormal autophagy, whereas under low-dose, controlled conditions, they may provide some experimental evidence for myocardial regeneration and repair in DCM by promoting the cardiomyogenic differentiation of stem cells.

Currently, no clinically validated dosage range or safety threshold has been established for cardiac treatments based on AgNPs. Healthy volunteers can tolerate short-term oral exposure to 100–480 µg of silver daily for 14 consecutive days; however, intravenous animal studies have shown that following administration of a 10 µg/mL solution, AgNPs accumulate in organs in a manner dependent on the formulation, particle size, and dose, and induce inflammation. Currently, 2.5 µg/mL is generally considered the in vitro proof-of-concept value [83,84].

#### 4.1.7. Silica Nanoparticle Therapy for DCM

Due to their structural stability, tunable porosity, and ease of surface modification, silica nanoparticles (SiNPs) are considered important candidate carriers for cardiovascular drug delivery; however, their application in DCM requires careful balancing of “cardiac-targeted delivery benefits” and “potential myocardial toxicity” [85]. The cardiac cell membrane-coated mesoporous silica nanoparticles (cCMCMSNs) developed by Pikwong et al. enhance the selective uptake of MSNs by cardiomyocytes and reduce phagocytosis by macrophages, demonstrating good potential for cardiac-targeted delivery [86].

Lozano et al. found that SiNPs can impair myocardial mitochondrial function, induce ROS production, and open the mitochondrial permeability transition pore (mPTP), leading to reduced ATP production, cardiomyocyte necrosis, and impaired cardiac mechanical function. These findings suggest that SiNPs may exacerbate DCM-associated systolic dysfunction via the “ROS-mPTP-mitochondrial energy depletion” pathway [87]. Further studies have demonstrated that SiNPs can induce pyroptosis in cardiomyocytes via the ROS/NLRP3/Caspase-1/GSDMD pathway and upregulate markers of cardiac hypertrophy such as ANP, BNP, and β-MHC, suggesting that they may be involved in inflammatory cardiac remodeling during the progression of DCM [88].

The toxicity of SiNPs primarily stems from their exposed silanol groups, residual synthetic reagents, and incomplete degradation or clearance. These characteristics can disrupt cell membranes, promote ROS production, damage mitochondria, reduce ATP synthesis, and activate NLRP3-mediated inflammation and pyroptosis. The degree of toxicity depends on factors such as particle size, shape, porosity, dose, exposure time, and route of administration. To determine whether a substance is harmful to the body, it is necessary to evaluate dose- and time-dependent parameters such as cell viability, membrane integrity, histopathology, biodistribution, degradation, and clearance rate. Only when these parameters meet the required standards can the substance be used in experiments.

#### 4.1.8. Iron Oxide Nanoparticle Therapy for DCM

Research involving iron oxide nanoparticles in the treatment of diseases serves mainly two separate functions: evaluating diseases through magnetic resonance imaging (MRI) and facilitating experimental therapy. Imaging probes utilize factors such as superparamagnetism, MRI contrast efficacy, macrophage uptake, and tissue distribution [89]. Lagan et al. employed ultrafine superparamagnetic iron oxide nanoparticles, alongside multi-sequence and multi-time-point MRI techniques, to illustrate that these nanoparticles are absorbed by cardiac macrophages and dispersed throughout the myocardial stroma. The R2*/R1 ratio acts as a quantitative measure of active infiltration by myocardial macrophages, reinforcing their potential as imaging probes for detecting myocardial inflammation in DCM [90].

The assessment of therapeutic applications takes into account factors such as bioactivity, safety related to dosage, tissue exposure, and the extent of recovery from cardiac damage. In a rat model of DOX-induced chemotherapy-associated cardiomyopathy, iron oxide nanoparticles (FeONPs) were found to lower levels of CK-MB, cTn-I, NADPH oxidase, and malondialdehyde (MDA); while enhancing the activity of superoxide dismutase (SOD) and glutathione peroxidase (GPx); and reducing inflammation, apoptosis, and damage to myocardial tissue. Moreover, the impact of FeONPs was further amplified when they were administered alongside selenium nanoparticles [91].

#### 4.1.9. Other

Protein NPs, nanocrystalline cellulose composite carriers, and collagen nanofiber scaffolds demonstrate potential for DCM-targeted treatment, myocardial toxicity protection, and tissue repair. Tomar et al. developed α-lactalbumin nanoparticles (LANPs) to deliver GFP-p53 DNA and carvedilol (CVD). LANPs loaded with GFP-p53 DNA and CVD formed spherical NPs, enabled efficient payload loading, and preferentially accumulated in the heart. In vivo, GFP-p53-DNA-LANPs produced stronger therapeutic effects than soluble GFP-p53-DNA, soluble CVD, or CVD-LANPs, restoring R-wave amplitude, R–R interval, and heart rate and improving myocardial structure, thereby providing a biocompatible, heart-targeted nanocarrier platform for gene and drug delivery [92]. Karimian et al. designed folate-targeted AF-NCC/Fe_3_O_4_ NPs as a DOX delivery vehicle. This nanosystem showed high encapsulation efficiency and acid-responsive drug release, enhancing antitumor activity while reducing toxicity to vital organs such as the heart, thereby offering a strategy to prevent chemotherapy-induced DCM [93].

Unlike systemically administered nanoparticles, collagen nanofibrous scaffolds are implantable tissue-engineering platforms that provide structural and biological support for myocardial repair. Joanne and colleagues created a clinical-grade scaffold made of collagen nanofibers, which was seeded with cardiomyocytes derived from human induced pluripotent stem cells. In a mouse model exhibiting nonischemic dilated cardiomyopathy (DCM), the implantation of this scaffold resulted in the stabilization of left ventricular volumes, preservation of left ventricular ejection fraction (LVEF), an increase in endothelial cell density, enhanced vascularization of the scaffold, and a decrease in fibrotic remodeling. Although the transplanted cells were no longer observable 14 days post-implantation, the functional advantages remained, reinforcing the potential applications of collagen nanofibrous scaffolds in cardiac tissue engineering, as opposed to systemic drug delivery [94]. Overall, nanoparticle delivery systems can improve the efficiency of payload delivery or reduce cardiotoxicity, while collagen nanofiber scaffolds promote myocardial repair through implantable tissue engineering methods. The general applicability of these strategies across different DCM subtypes remains uncertain. Certain miRNA profiles may be associated with multiple etiologies. Future research should further clarify the different subtypes to provide more precise guidance for clinical treatment. We have summarized the mechanisms by which various NPs treat DCM in Table 2.

### 4.2. Nanoparticle Therapy for HCM

#### 4.2.1. PLGA Therapy for HCM

Melatonin has potential anti-hypertrophic and anti-fibrotic effects, but its short residence time in the cardiac microenvironment limits its therapeutic application [95]. Zhao et al. developed a dual-targeting nanoplatform in which melatonin and superparamagnetic iron oxide nanoparticles (SPIONs) were encapsulated in PLGA-COOH nanoparticles functionalized with a cardiac homing peptide (CHP). CHP provided molecular targeting of the hypertrophied myocardium, whereas the superparamagnetic property of SPIONs enabled external magnetic field-guided cardiac accumulation. In the presence of an external magnetic field, the nanoparticles showed greater myocardial accumulation and enhanced the therapeutic effects of melatonin, reducing cardiac hypertrophy, fibrosis, and the expression of collagen I, TGF-β1, and Smad3 [96].

#### 4.2.2. Hydrogels Therapy for HCM

Nanohydrogels are three-dimensional polymer networks formed by physical or chemical cross-linking of polymers such as polyethylene glycol and hyaluronic acid. They are widely used in drug delivery, bioimaging, tissue engineering, and flexible electronic devices, for example, in the targeted controlled release of anticancer drugs and the performance enhancement of conductive hydrogel sensors. Incorporating nanostructures into hydrogels can enhance mechanical strength, drug delivery, and biocompatibility [97]. Zhao et al. developed an injectable composite hydrogel encapsulating doxorubicin and hydroxycamptothecin for the injectable, dual-stimulus-responsive drug release in tumors. The hydrogel demonstrated extremely high photothermal conversion efficiency and emerged as a potential universal carrier for the localized delivery of multiple drugs, offering a potential approach to mitigating chemotherapy-related cardiotoxicity [98].

Hydrogels have garnered increasing attention in recent years as drug-delivery platforms for cardiovascular diseases. However, direct evidence supporting the use of therapeutic nanohydrogels for primary HCM remains limited. Most hydrogel studies related to HCM have utilized these materials as three-dimensional scaffolds or mechanically tunable matrices to replicate the myocardial microenvironment and investigate disease mechanisms, rather than as therapeutic nanoparticles. Although these studies have demonstrated the therapeutic potential of nanocarriers and hydrogel platforms in pathological cardiac remodeling, their efficacy in sarcomeric or other forms of primary HCM remains a topic for further exploration.

### 4.3. Nanoparticle Therapy for Diabetic Cardiomyopathy

#### 4.3.1. PLGA Therapy for Diabetic Cardiomyopathy

PLGA NPs can influence the treatment of diabetic cardiomyopathy through various mechanisms, including oxidative stress reductions in cardiomyocytes, thereby protecting the heart muscle. Zhang et al. used novel PLGA-GLP-1 NPs with poly (lactic acid) as the drug carrier to investigate the protective effects of these NPs against myocardial damage in diabetic rats. The results indicated that PLGA-GLP-1 NPs can enhance the antioxidant capacity of cardiomyocytes while promoting mitochondrial DNA in cardiomyocytes, the PLGA-GLP-1 nanoparticles prepared in this study had a diameter of approximately 40–60 nm. Degradation accelerated after 90 days [99]. Additionally, a PLGA-loaded nanoemulsion of the sodium-glucose cotransporter-2 (SGLT-2) inhibitor dapagliflozin (DAPA) was used to treat AC16 cells from mice with diabetic cardiomyopathy. Compared to the control group, PLGA-loaded treatment reduced the levels of oxidative stress-related markers in mouse myocardial tissue, while also protecting mitochondrial function and regulating myocardial iron metabolism to treat diabetic cardiomyopathy and the resulting heart failure [100]. GAO et al. also used polysaccharide sulfate -loaded PLGA NPs to treat coronary microcirculatory dysfunction in diabetic cardiomyopathy, restoring cardiac function in rats via the NF-κB-PEGA pathway, while simultaneously reducing inflammation and oxidative stress levels [101].

PLGA has also contributed to diabetic cardiomyopathy diagnosis and monitoring. Gao et al. used the double-emulsion evaporation method to prepare perfluoropropane (C3F8) and PLGA nanobubbles coated with fibroblast growth factor 21 (FGF21). These nanobubbles can be monitored by ultrasound imaging and downregulate apoptosis-related CTGF and caspase-3 mRNA in cardiomyocytes, thereby inhibiting pathological damage such as cellular hypertrophy caused by diabetic cardiomyopathy [102].

#### 4.3.2. Chitosan Therapy for Diabetic Cardiomyopathy

Chitosan is a natural, safe, semi-crystalline cationic polymer obtained by deacetylation of chitin. It is the second most common copolymer composed of (1-4)-2-amino-2-deoxy-D-glucan and is widely used due to its excellent biocompatibility, biodegradability, abundance, adaptability, and low toxicity. One of the most well-known applications of chitosan-based biomaterials in tissue regeneration is cardiac tissue engineering, which offers properties such as antioxidant, anti-inflammatory, antibacterial, anti-obesity, hypoglycemic, and antihypertensive effects. As a biodegradable nanocarrier, chitosan can improve drug encapsulation, stability, and controlled-release properties. Although it may possess antidiabetic and anti-inflammatory activity on its own, these effects should be distinguished from those of the encapsulated drug through appropriate blank carrier controls [103].

Chitosan has demonstrated potential for diabetic cardiomyopathy treatment. Georgică et al. compared the efficacy of chitosan and dapagliflozin in mice with diabetic cardiomyopathy using echocardiography assessment and found that chitosan has a better therapeutic effect on diabetic cardiomyopathy [104]. Metformin is the most commonly used drug for treating diabetes. Combination therapy with metformin and chitosan NPs produces stronger antidiabetic effects, including reduced fasting blood glucose and insulin levels. Chitosan NPs also exerted significant effects on the apoptotic proteins Bax, Caspase-3, Fas, and Fas-L, while simultaneously reducing inflammatory cytokine levels and restoring the antioxidant capacity of cardiomyocytes [105].

#### 4.3.3. EVs Therapy for Diabetic Cardiomyopathy

Several reviews have summarized EV research in diabetic cardiomyopathy. A 2024 article explores the therapeutic potential of EVs for diabetic cardiomyopathy from multiple perspectives, including endothelial injury and myocardial hypertrophy, and the present review supplements this work [106]. Adipose-derived stem cell-derived EVs (ADSC-EVs) have anti-inflammatory and immunomodulatory effects. Zhang et al. administered ADSC-EVs to diabetic cardiomyopathy models in mice and cells and investigated their mechanisms. They identified Chit1 through gene sequencing and validated the therapeutic effects through relevant signaling pathways. ADSC-EVs alleviated diabetic cardiomyopathy by downregulating the NLRP3/caspase-1 pathway through Chit1 [107].

However, the release of EVs also has adverse effects. Studies have confirmed that hyperglycemia can damage mitochondria via the SIRT1/AMPK/PGC1α signaling pathway, leading to cardiomyocyte apoptosis and the release of extracellular vesicles containing mitochondrial α, thereby exacerbating the progression of diabetic cardiomyopathy [108].

#### 4.3.4. Gold Nanoparticle Therapy for Diabetic Cardiomyopathy

AuNPs may treat early diabetic cardiomyopathy-associated left ventricular hypertrophy and wall dysfunction by reducing the left ventricular hypertrophy index and lowering vascular endothelial growth factor-A (VEGF-A) and TGF-β mRNA expression in myocardial tissue [109]. Estrogen deficiency resulting from ovariectomy exacerbates cardiac inflammation in diabetic mice, manifested by a higher proportion of pro-inflammatory type 1 macrophages (M1) compared to anti-inflammatory type 2 macrophages (M2). Intravenous injection of AuNPs containing miR-155 into the rat tail vein restores cardiac function, increases the proportion of M2 macrophages, and reduces ROS levels, The 10 nm AuNP cores were conjugated with thiol-modified antago-miR-155 through Au–S bonds, resulting in conjugates of approximately 20–40 nm. Their preferential macrophage uptake was attributed to scavenger receptor-associated phagocytosis rather than an additional targeting ligand [110]. Gold nanoclusters coated with negatively charged polyvinylpyrrolidone (PVP) have also been shown to inhibit the overexpression of mitochondrial ROS while stabilizing the mitochondrial membrane potential of cardiomyocytes. These studies collectively demonstrate the potential of AuNPs in the treatment of diabetic cardiomyopathy [111].

Curcumin, a natural polyphenolic compound extracted from turmeric rhizomes, has several biological activities relevant to the treatment of DCM, including regulating autophagy, ferroptosis, oxidative stress, inflammation, and pyroptosis; however, its efficacy is limited by poor water solubility and biocompatibility [112]. The use of gold nanoclusters can enhance the bioavailability of curcumin and reduce intracellular ROS levels and lipid accumulation in diabetic cardiomyopathy cells, thereby treating the condition by downregulating PPARα expression [113].

#### 4.3.5. Silver Nanoparticle Therapy for Diabetic Cardiomyopathy

AgNPs are widely used in biomedicine due to their antibacterial, antiviral, and anticancer properties, relative biocompatibility, and size-dependent ability to inhibit ROS production, DNA damage, and enzyme activity. These mechanisms suppress microbial growth and replication [114]. Atale et al. synthesized AgNPs with an average particle size of 40–100 nm using Syzygium cumini, an ethanol extract from dietary cumin seeds. Treatment of H9C2 cardiomyocytes with these AgNPs restored cell morphology and reduced lipid peroxidation products, indicating suppression of the cardiac stress response [115].

#### 4.3.6. Carbon Nanoparticle Therapy for Diabetic Cardiomyopathy

Carbon nanomaterials are a key component of the field of nanomaterials, primarily including carbon nanotubes, fullerenes, graphene, nanodiamonds, and their derivatives. They are widely used in various biomedical fields, such as drug and gene delivery, bioimaging, cancer therapy, antiviral and antibacterial applications, and biosensing [116].

Current applications of carbon NPs in diabetic cardiomyopathy is primarily focused on early detection. MRI is a powerful tool for detecting cardiac abnormalities, and ROS are early biomarkers of diabetic cardiomyopathy. Nie et al. used fluorocarbon nanosheets containing polyhydroxy groups and 2,2,6,6-tetramethylpiperidine-1-oxy to prepare an ROS-specific MRI nanoprobe for early In vivo detection of diabetic cardiomyopathy [117]. Another study developed a water-soluble electrochemiluminescence biosensor based on carboxylic acid-rich γ-C3N4 nanoparticles (CNNP), which can monitor ROS levels and metabolic function in cardiomyocytes for early detection [118].We have summarized the mechanisms by which various nanoparticles treat diabetic cardiomyopathy in Table 3.

### 4.4. Nanoparticle Therapy for ACM

ACM is characterized by progressive loss of cardiomyocytes and replacement by fibro-adipose tissue. Lin evaluated the efficacy of EVS in a mouse model of ACM and found that mice treated with EVS exhibited improved cardiac function, reduced expression of the pan-inflammatory transcription factor NF-κB and NLRP3, and fewer arrhythmias [119]. To treat arrhythmias and induced cardiomyopathy caused by hiPSC-CMs, Richards et al. introduced conductive silicon nanowires into hiPSC cardiac spheroids. By improving the endogenous electrical microenvironment, this approach provides an advanced cell delivery tool for next-generation cardiac repair [120].

### 4.5. Plant-Derived Nanoparticle Therapy for Cardiomyopathy

Over the past 70 years, plant extracts and natural herbal medicines have attracted research interest due to their anti-inflammatory and anti-allergic properties and minimal side effects [121]. Many plant-derived bioactive compounds may also act as chemopreventive agents for cardiomyopathy, but enzymatic degradation and low bioavailability limit their application. NPs can protect active ingredients from degradation and improve solubility [122]. In addition, plant-derived compounds may exert multi-target biological effects and enhance the activity of conventional drugs. Emodin can disrupt bacterial membrane integrity, inhibit bacterial biofilm formation, and produce synergistic effects with β-lactam antibiotics, suggesting its potential as a bioactive carrier in combination therapy strategies [123].

Ligustrazine (LIG) is a bioactive alkaloid extracted from the rhizomes of the medicinal plant Ligusticum chuanxiong. It possesses cardioprotective effects, but poor water solubility and low bioavailability. Wang et al. used a tetrahedral-framework nucleic acid molecule as a nanocarrier to treat doxorubicin-induced cardiomyopathy (DIC). This nanocarrier improved myocardial injury by inhibiting mitochondrial homeostasis disruption [124]. Tohidi et al. also studied DIC and found that nano-curcumin capsules reduce doxorubicin-induced cardiotoxicity [125].

Hanafy et al. developed starch-albumin hydrogel NPs containing anthocyanins as the active ingredient. By leveraging the size effect and structural barriers of the nanocarriers to enhance cardiac retention, they targeted repair of carbon tetrachloride-induced myocardial fibrosis and glycogen storage cardiomyopathy. The NPs restored myocardial structure and metabolic homeostasis by scavenging lipid peroxidation products and correcting abnormal myocardial glycogen deposition [126]. Hydrogel platforms can also enhance the delivery of plant-derived bioactive compounds. A transdermal hydrogel loaded with steam-extracted Panax notoginseng saponins promoted wound healing through anti-inflammatory and regenerative effects. This provides a useful reference for improving the topical delivery and therapeutic activity of plant-derived compounds [127]. Mageid et al. used nano-curcumin and aged garlic extract to treat streptozotocin-induced diabetic cardiomyopathy. Nano-curcumin alleviated oxidative stress and advanced glycation end product (AGEs)-related damage by upregulating myocardial Mn-SOD and downregulating RAGE expression, whereas aged garlic extract exerted hypoglycemic, anti-inflammatory, and antioxidant effects. Both agents reduced myocardial enzyme levels and improved myocardial pathological damage and fibrosis [128]. These studies show that nanoparticle-encapsulated drugs enhance myocardial anti-inflammatory and antioxidant capacity, thereby improving cardiac function. We have summarized the mechanisms by which various plant-derived nanoparticles treat cardiomyopathy in Table 4.

## 5. Targeted Delivery of Nanomedicines in the Treatment of Cardiomyopathy

Passive targeting is simple to prepare and easy to translate into clinical practice, but it relies on the EPR effect, has poor specificity, and tends to accumulate in the liver and kidneys; active targeting offers precise lesion recognition and lower off-target toxicity, but is associated with immunogenicity and challenges in translation. Therefore, different delivery strategies must be used for different drugs to achieve the best therapeutic outcomes.

### 5.1. Passive Targeting

The tissue distribution of nanomedicines is influenced by vascular permeability. In tumors, abnormal vascular structures and impaired lymphatic drainage jointly promote the extravasation and retention of nanoparticles, resulting in the classic enhanced permeability and retention (EPR) effect. Myocardial ischemia, infarction, and inflammation similarly disrupt endothelial integrity and increase vascular permeability to macromolecules and nanoparticles [129,130]. However, existing cardiac studies primarily demonstrate vascular leakage and localized nanoparticle accumulation, rather than long-term retention resulting from impaired lymphatic drainage. Therefore, the process of nanoparticle delivery to damaged myocardial tissue is more accurately described as accumulation mediated by vascular permeability, rather than the classic EPR effect. Liposomes, polymeric nanoparticles, and exosomes can deliver anthocyanins, curcumin, growth factors, and other therapeutic agents to alleviate oxidative stress, inflammation, fibrosis, and ferrocytosis, thereby improving myocardial remodeling and cardiac function. Hydrogels directly injected into myocardial tissue can also prolong local drug retention time; however, their primary role is as local delivery carriers, rather than as nanoparticles that accumulate via increased vascular permeability. Passive targeting relies on nanocarrier physicochemical properties, including particle size, surface characteristics, and nonspecific interactions, to facilitate preferential accumulation and retention within diseased tissues, thereby enhancing local therapeutic exposure [131].

Mitochondrial dysfunction is a hallmark of early-stage diabetes [132,133]. Wang et al. developed a system using cationic microbubbles (CMBs) to deliver RORα plasmids, combined with ultrasound-targeted microbubble disruption (UTMD) technology, for the treatment of sepsis-induced cardiomyopathy. The microbubbles passively targeted and accumulated in the damaged myocardial regions, where they released the plasmids at specific sites under ultrasound stimulation, thereby achieving RORα-specific overexpression in the myocardium. The results demonstrated that this delivery system significantly enhances the cardioprotective effects of melatonin [134]. Guo developed macrophage-membrane-coated PLGA nanoparticles (MGP) to deliver GDF15 for the treatment of septic cardiomyopathy by targeting macrophages. The nanocarriers were not modified with specific ligands; instead, they relied on the EPR effect in inflamed myocardium to achieve passive targeting and accumulation, while their biomimetic cell membrane properties extended their circulation in vivo. By inhibiting NLRP3 inflammasome activation through the GDF15-MYPT1-AKT-YBX1 pathway, the treatment significantly improved cardiac function and reduced myocardial damage [135]. Furthermore, multiple studies have used NPs without specific ligand modification, relying on the EPR effect to accumulate in diseased myocardium and target cardiomyocytes. By delivering growth factors, siRNA, or antioxidants, these NPs effectively improve various types of myocardial injury, providing a safe and simple passive targeted nanotherapy strategy for cardiomyopathy [136,137,138]. We have summarized the particles, ligands, target organs, and mechanisms involved in passive targeted delivery for cardiomyopathy in Table 5.

### 5.2. Active Targeting

Active targeting modifies nanocarrier surfaces with specific ligands, targeting peptides, antibodies, or receptor substrates to enable ligand-receptor-mediated delivery to target cells or tissues. This approach improves targeting accuracy, selectivity, and intracellular delivery efficiency. Active-targeting nanosystems can recognize pathological cells, including cardiac fibroblasts and macrophages, enhancing drug accumulation and uptake in diseased heart tissue while reducing systemic exposure and toxicity [139]. Du et al. developed a dual-targeted nanoplatform (ESC-AGO-2) aimed at diabetic cardiomyopathy, which is founded on a minuscule iron oxide core that has been functionalized with a cardiac-homing peptide (CHP) alongside the mitochondrial-targeting peptide SS-31. The CHP enhanced the accumulation of nanoparticles in the damaged myocardium, while SS-31 facilitated their later localization to the mitochondria, thus allowing for a tiered delivery of AGO-2 to the cardiac mitochondria. This approach reinstated mitochondrial equilibrium and diminished oxidative stress as well as inflammation [140].

Zhao et al. developed a peptide-conjugated nanoprobe specifically targeting primary cardiomyocytes (PCMs) by incorporating perfluorocarbon (PFP) as the core and loading it with estradiol (E2). This nanoprobe achieves active targeting through the specific binding of the PCM-specific peptide to cardiomyocytes, enabling precise recognition and binding to cardiomyocytes, thereby enhancing the accumulation of estradiol in the heart and effectively inhibiting cardiac hypertrophy and fibrosis. Accumulation within the myocardium was still observable 12 h after administration, and the circulating signal persisted for more than 24 h [141]. Sepsis-associated cardiomyopathy is a serious complication of sepsis associated increased patient mortality. Researchers have developed a chromium-based nanoenzyme capable of precisely targeting cardiac muscle mitochondria, promoting the decomposition of ROS, inhibiting iron-dependent cell death, and reducing lipid peroxidation [142]. Additionally, Zeng et al. designed baicalin nanofibers that target the angiotensin II type I receptor. By reducing hydrogen peroxide accumulation and inhibiting ferroptosis, these nanofibers improved the survival of rat cardiomyocytes in DIC model, in H9c2 cells, AT1R expression increased approximately 1.8-fold, and binding of BA-NF was observed after 1 h [143].

**Figure 3:**
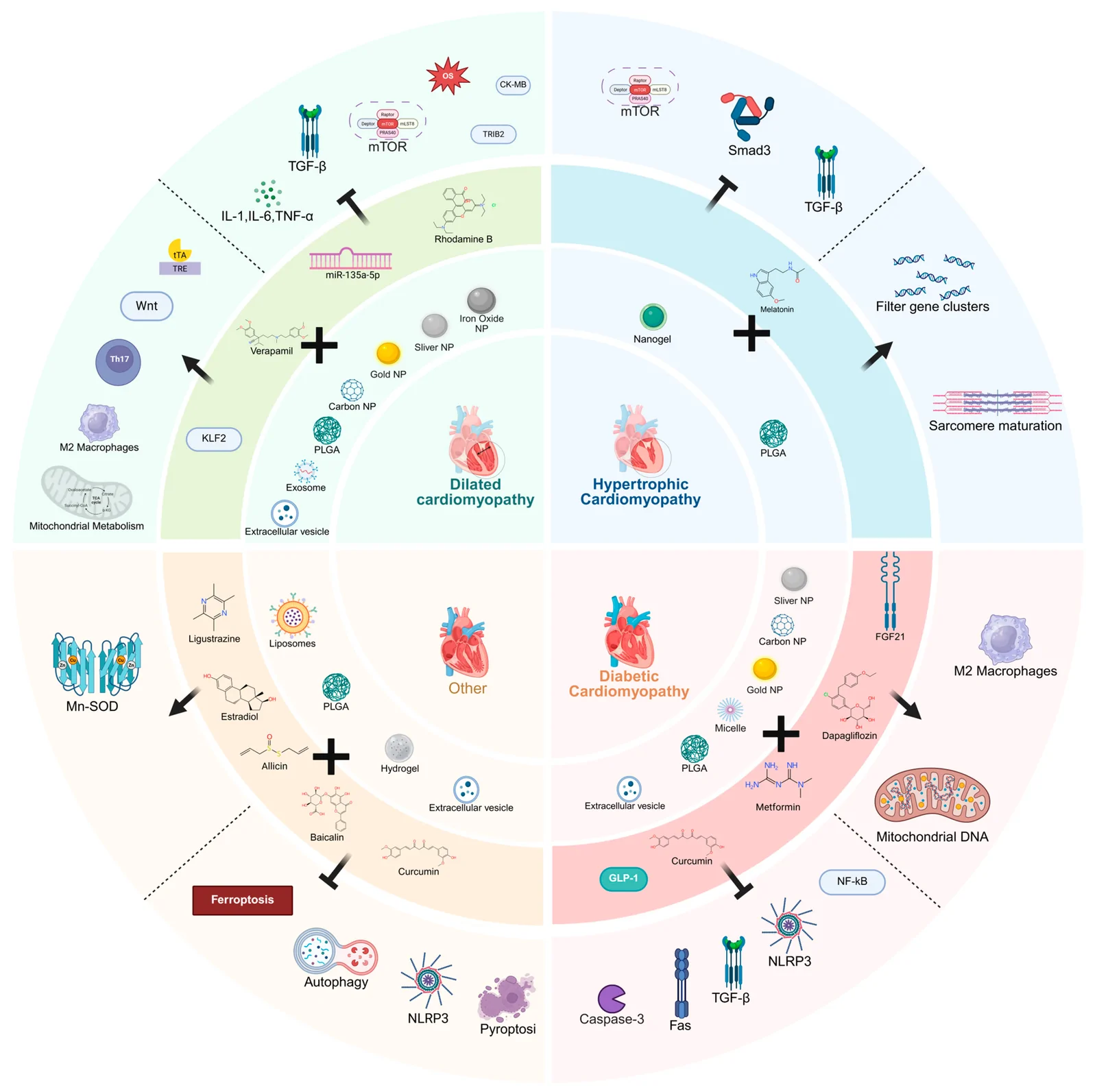
Different nanoparticles can intervene in cardiomyopathy through different mechanisms. PLGA, poly(lactic-co-glycolic acid); CK-MB, creatine kinase mb isoenzyme; OS, oxidative stress; mTOR, mechanistic target of rapamycin; TRB2, t-box transcription factor 2; TGF-β,transforming growth factor beta; IL-1,interleukin-1; IL-6,interleukin-6; TNF-α, tumor necrosis factor alpha; tTA,tetracycline-controlled transactivator; TRE, tetracycline response element; Wnt, wingless-related integration site; Th17, t helper 17 cells; Smad3, sma- and mad-related protein 3; TGF-β, transforming growth factor beta; NF-kB, nuclear factor kappa B; NLRP3, nlr family pyrin domain containing 3; Fas, fas cell surface death receptor; Caspase-3, cysteine-aspartate protease 3; Mn-SOD, manganese superoxide dismutase.

Since inflammation-induced cardiac fibrosis can progress to DCM, and cardiac fibroblasts play a key role in this process, Shen et al. designed a fibroblast-targeted biomimetic nanoparticle; the results showed that this nanoparticle significantly reduced myocardial inflammatory responses and inhibited myocardial fibrosis. Mlipo@MLN4924, utilizing fibroblast membrane-mediated homotargeting, increased its accumulation in the heart by approximately 2.5-fold by the 24th hour [144]. We summarized the particles, ligands, target organs, and mechanisms involved in active targeted delivery for cardiomyopathy in Table 6, and summarized the mechanisms mentioned throughout this paper in Figure 3.

## 6. Conclusions and Challenge

This review summarizes recent advances in nanoparticle-based treatment of cardiomyopathy. These NPs may improve myocardial drug delivery and exert cardioprotective effects by modulating oxidative stress, inflammation, mitochondrial dysfunction, regulated cell death, fibrosis, and myocardial remodeling.

Further research is needed to advance the translational application of NPs in the field of cardiomyopathy. Current research has primarily focused on diabetic cardiomyopathy, highlighting the need to explore other subtypes of cardiomyopathy. Furthermore, most of the existing evidence remains at the preclinical stage, with very few clinical studies; no large-scale, multicenter Phase III clinical trials have yet been conducted. Clinical translation is further limited by variable in vivo stability and biodistribution, off-target accumulation, immunotoxicity and chronic toxicity, disease heterogeneity, interspecies differences, and high research and development costs. Future research should prioritize nanocarriers with high biocompatibility and low toxicity, biomimetic and stimulus-responsive designs, and the optimization of targeting ligands and physicochemical properties to enhance myocardial accumulation, cellular uptake, and retention rates. Combining nanodelivery with gene therapy, stem-cell therapy, and tissue engineering may further enable multimodal and synergistic therapeutic strategies. Most importantly, systematic solutions are required to overcome the barriers between preclinical development and clinical application.

To address these translational limitations, standardized methods should be established to evaluate nanoparticle stability, targeting performance, biodistribution, efficacy, and long-term safety. Human-relevant cardiac models and large-animal studies may improve the prediction of clinical responses. Reproducible manufacturing, quality control, and well-designed clinical trials are also essential for identifying nanotherapeutics with genuine clinical potential. Beyond these considerations, from a broader perspective, future research should shift from demonstrating the feasibility of nanoparticles to establishing disease-specific, clinically validated therapeutic paradigms. For example, cardiomyopathy is not a single biological entity; even within the same clinical subtype, patients may exhibit significant differences in genetic background, inflammatory activity, metabolic dysfunction, degree of fibrosis, and disease stage. Therefore, future nanotherapeutics should be developed based on molecular phenotypes and therapeutic windows, rather than solely on traditional diagnostic categories. Biomarkers capable of identifying dominant pathological mechanisms, predicting nanoparticle accumulation in the myocardium, and monitoring target binding—or serving as early diagnostic tools—are crucial for selecting patients most likely to benefit. Human-relevant experimental systems—including patient-derived induced pluripotent stem cell-derived cardiomyocytes, cardiac organoids, engineered cardiac tissue, and organ-on-a-chip platforms—help bridge interspecies differences and more accurately predict efficacy, pharmacokinetics, and toxicity prior to clinical trials. This enables nanomedicine to evolve from an experimental drug-delivery technology into a precision therapy strategy capable of modulating disease progression and improving the long-term prognosis of various cardiomyopathy subgroups defined at the molecular level.

In summary, nanomaterials offer new therapeutic approaches for cardiomyopathy through their physicochemical properties, biocompatibility, targeted delivery capacities, and reparative effects. They address limitations of conventional drug delivery and support precise targeting, efficient delivery, and safe tissue repair. Although challenges remain, integration of material science, biomedicine, nanotechnology, and translational medicine may allow nanomaterials to play a central role in the precise diagnosis, targeted treatment, tissue repair, and prognosis of cardiomyopathy, ultimately benefiting patients with cardiomyopathy.

## Figures and Tables

**Table 1:** Advantages and limitations of commonly used inorganic NPs.

No.	NPs	Advantages	Limitations	Ref.
1	Carbon-based NPs	High surface area, good drug-loading capacity, easy functionalization, and useful optical and electrical properties	Poor biodegradability, aggregation, oxidative stress, inflammation, and potential pulmonary toxicity	[56]
2	Gold NPs	Good stability, easy surface modification, and optical properties	Poor biodegradability, hepatotoxicity	
3	Silver NPs	Antimicrobial, anti-inflammatory, and easily functionalized	Ag^+^ release may induce oxidative stress, inflammation, DNA damage,	
4	Silica NPs	High surface area, adjustable pore size, and high drug-loading capacity	Slow degradation and possible accumulation in the liver, spleen, and lungs	
5	Iron oxide NPs	Magnetic targeting, MRI capability, and controllable surface modification	Excess iron may promote ROS production, iron overload, and hepatic or renal injury	

**Table 2:** Potential Applications of Nanoparticles in the Treatment of DCM.

No.	Drug	NPs	Nanoparticle Parameters				Study Design	Therapeutic Schedule	Administration	Pathway	Effect	Ref.
			Size	Zeta Potential	PDI	Drug Loading/EE						
1	-	Exosomes	30–100 nm	-	-	-	In vitro: hiPSC-CMs	0.5–4% (*v*/*v*) exosome	Cell incubation, 72 h	-	Induces pathological myocardial remodeling	[63]
2	-	Mesenchymal stem cell-derived exosomes	35.21 nm	-	-	-	In vitro: NMVMs; In vivo: C57BL/6 mice	300 μg in 200 μL PBS	Tail-vein	JAK2/STAT6	Reduces myocardial apoptosis, inflammation, and M1 macrophage polarization	[65]
3	let-7i	Trophoblast stem cell-derived exosomes	50–150 nm	-	-	-	In vitro: AC16 cells; In vivo: C57BL/6 mice	100 µL TSC-Exos	Multipoint intramyocardial injection	let-7i/YAP	Reduces cardiomyocyte apoptosis, inflammation, fibrosis, and cardiac dysfunction	[66]
4	-	Cardiac stem cell-derived exosomes	129.6 nm	-	-	-	In vitro: NRCMs; In vivo: CD-1 mice	Single dose of 30 × 10^9^ particles in 200 μL PBS	Tail-vein	-	Improves cardiac function and reduces myocardial apoptosis and fibrosis	[67]
5	Lipo@miR-185-5p inhibitor	liposomes	172.35 ± 0.8 nm	+29.7 mV	<0.15	EE: 98.0 ± 1.2%	In vitro: H9C2 and hiPSC-CMs; In vivo: C57BL/6 mice	5 mg/kg (0.1 mg/mouse in 100 μL)	Tail-vein	BCL-2-BAX	Improves cardiac function and reduces apoptosis, oxidative stress, fibrosis, and DNA damage	[68]
6		KLF2-overexpressing endothelial cell-derived EVs	100–150 nm	-	-	-	In vivo, C57BL/6 mice	100 µg	Tail-vein, twice	CCR2/Ly6C^high^ monocyte mobilization	Reduces ventricular remodeling, myocardial inflammation, fibrosis, and monocyte recruitment	[72]
7	-	Ag-NPs	50–80 nm	-	-	-	In vitro, BM-MSCs,	2.5 µg/mL	Cell incubation, 14 days	Wnt3/β-catenin	Promotes myocardial differentiation of bone marrow mesenchymal stem cells and telomere/TERT-related regeneration	[82]
8	Rhodamine B	cCMCMSNs	139.4 ± 12.8 nm	−32.16 ± 0.60 mV	0.54 ± 0.06	EE: 94.71 ± 1.01%	In vitro: AC16, A549, HepG2 and RAW264.7 cells	25 µg/mL	Cell incubation, 4 h treatment	Homotypic cardiac targeting and immune escape	Enhances uptake by cardiac cells, reduces phagocytosis by macrophages, and exhibits low cytotoxicity toward cardiomyocytes	[86]
9		FeO NPs, Se^6+^ NPs	FeO: 122.3 ± 29.3 nm; Se^6+^: 105.3 ± 30.09 nm	FeO: +37.5 ± 4.56 mV; Se^6+^: +34.7 ± 4.23 mV	-	-	In vivo, Wistar rats	0.5 mg/kg	Intraperitoneal, 3 weeks	Nox1/p53	Reduces oxidative stress, inflammation, and apoptosis in cardiac muscle cells	[91]

PLGA, poly(lactic-co-glycolic acid); DOX, doxorubicin; iPSC-CM, human induced pluripotent stem cell-derived cardiomyocyte; BCL-2, B-cell lymphoma/leukemia-2; BAX, Bcl-2-associated X protein; mTOR, mechanistic target of rapamycin; TGF-β1, transforming growth factor beta 1.

**Table 3:** Potential Applications of Nanoparticles in the Treatment of Diabetic Cardiomyopathy.

No.	Drug	NPs	Nanoparticle Parameters				Study Design	Therapeutic Schedule	Administration	Pathway	Effect	Ref.
			**Size**	**Zeta Potential**	**PDI**	**Drug Loading/EE**						
1	Glucagon-like peptide 1	PLGA	40–60 nm	−6.99 mV	-	-	In vivo, Wistar rats, High-sugar diet model	1.0 mg/kg or 2.0 mg/kg or 3.0 mg/kg, intraperitoneal injection	Oral	Bcl-2/Bax	Enhances antioxidant capacity, inhibits cardiomyocyte apoptosis, and promotes mitochondrial DNA repair	[99]
2	Dapagliflozin	PLGA	66.1 nm	+16.5 mV	-	Drug loading: 3.82 ± 2.11%; EE: 86.11 ± 0.49%	In vivo, C57BL/6J mice, Streptavidin model	20 mg/kg/day by gavage	Oral 6 weeks	Tfr1	Inhibits myocardial fibrosis and oxidative stress, protects mitochondrial function, and regulates myocardial iron metabolism	[100]
3	Polysaccharide sulfate	PLGA	180.6 nm	-	-	EE: 75.80%	In vivo, SPF rats, Streptavidin model	20 mg/kg/day, intraperitoneal injection	Intraperitoneal 3 days	NF-κB-PEGA	Alleviates diabetic cardiomyopathy and restoring cardiac function in rats via NF-κB signaling pathway	[101]
4	Fibroblast growth factor 21	PLGA	880 ± 20 nm	≈0 mV	Drug loading: 3.4 ± 1.9%; EE: 68.1 ± 3.8%		In vitro, H9C2 cell In vivo, C57/BL6, Streptavidin model	In vitro, 125 ng/mL In vivo, 100 μg/kg, slow intravenous administration	LFUS: 500 kHz, 2 W for 5 min Tail vein 8 weeks	Caspase-3	Reduced myocardial hypertrophy, apoptosis, and interstitial fibrosis	[102]
5	Metformin	Chitosan	6–22 nm	-	-	-	In vivo, Sprague Dawley, Streptavidin model	500 mg/kg body weight per/day	Oral 60 days	Bax-Caspase-3	Lowered fasting blood glucose, upregulated BCL-2 expression, and restored myocardial antioxidant capacity	[105]
6	-	Extracellular vesicles	30–1000 nm	-	-	-	In vitro, H9C2 cell In vivo, SD rats, Streptavidin model	In vitro, 25 ug/mL or 50 ug/mL 100 ug/mL, In vivo, 200 ug/kg	Tail-vein 4 weeks	NLRP3/Caspase-1	Downregulates NLRP3/caspase-1 to alleviate diabetic cardiomyopathy	[107]
7	-	AuNPs	50 ± 4.7 nm	−12.3 ± 3.7 mV	0.15	-	In vivo, SD rats, Streptavidin model	2.5 mg/kg/day	Intraperitoneal 7 weeks	VEGF-TGF-β	Lower blood glucose and TGF-β levels	[109]
8	Syzygium cumini	Silver NPs	43.02 ± 1.25 nm	−19.6 ± 0.5 mV	-	-	In vitro, H9C2 cell	Treat with the optimal dose of SmSNPs and glucose for 48 h	Substrate 48 h	-	Restores cellular morphology and suppresses lipid peroxidation, reducing cardiac stress	[115]

PLGA, poly(lactic-co-glycolic acid); BCL-2, B-cell lymphoma/leukemia-2; BAX, Bcl-2-associated X protein; NLRP3, NOD-like receptor protein; caspase-1, interleukin-1 p-converting enzyme; VEGF, vascular endothelial growth factor; TGF-β, transforming growth factor beta; NF-κB, nuclear factor kappa-B; ROS, reactive oxygen species.

**Table 4:** Potential Applications of Plant-Derived Nanoparticles in the Treatment of Cardiomyopathy.

No.	Drug	NPs	Nanoparticle Parameters				Study Design	Therapeutic Schedule	Administration	Pathway	Effect	Ref.
			**Size**	**Zeta Potential**	**PDI**	**Drug Loading/EE**						
1	Ligustrazine	Phospholipid-cholesterol nanovesicles	82.1 ± 0.25 nm	−0.249 ± 0.047 mV	0.21 ± 0.01	-	In vitro, HL-1 cell In vivo, C57BL/6 mice	In vitro, 25–100 μmol/L In vivo, 50 mg/kg/day	Intraperitoneal 14 days	PIEZO1-TMBIM6-–PHB2	Restored mitochondrial homeostasis, inhibited pyroptosis and fibrosis	[124]
2	Anthocyanins	Hydrogel	-	≈−30 mV	-	-	In vivo, mice	In vivo, 50 μg/kg, twice weekly for 3 weeks	Oral 3 weeks	-	Restores myocardial glycogen levels and improves myocardial pathological damage and cardiac function	[126]
3	Curcumin and aged garlic extract	Curcumin NPs	±200 nm	-	-	-	In vivo, SD rat, STZ model	In vivo, 300 mg/kg, aged garlic extract 500 mg/kg for 56 days	Oral 56 days	SOD-RAGE	Inhibits oxidative stress and advanced glycation end product-mediated damage and improves myocardial necrosis and fibrosis	[128]

NLRP3, NOD-like receptor protein; SOD, superoxide dismutase; RAGE, receptor for advanced glycation end products.

**Table 5:** Passive Targeted Delivery in Cardiomyopathy.

No.	Drug	NPs	Targeting Ligand	Target Organ	Nanoparticle Parameters				Targeted Cells	Study Design	Therapeutic Schedule	Administration	Pathway	Effect	Ref.
					Size	Zeta Potential	PDI	Drug Loading/EE							
1	GDF15	PLGA	No specific ligand	Heart/myocardium	≈290 nm	≈−11 mV	-	Drug loading: 0.724%; EE: 84.21%	Macrophages	In vitro, H9c2 cells; In vivo, C57BL/6J mice, LPS model	In vitro, 50 ng/mL In vivo, 0.1 mg/kg	Tail vein	GDF15-MYPT1-AKT-YBX1	Inhibits NLRP3, improves cardiac function, and reduces myocardial damage	[135]
2	bFGF	Poloxamer 188-grafted heparin copolymer	Passive myocardial accumulation assisted by UTMD	Heart/myocardium	128 ± 1.65 nm	−15.3 ± 1.6 mV	0.089	EE: 84.3 ± 2.8%	Microvascular endothelial cells	In vitro, H9c2 cells; In vivo, SD rats, STZ model	In vitro, 50 μg/mL In vivo, 3 μg/kg	UTMD: MI 1.9 for 10 s Tail vein on days 1, 3 and 5	bFGF-FGFR	Enhances cardiomyocyte uptake, improves cardiac function, and reduces myocardial collagen fraction	[137]
4	bFGF	Poloxamer 188-grafted heparin copolymer	Passive myocardial accumulation	Heart/myocardium	128 ± 1.65 nm	≈−15 mV	≈0.1	EE: 84.3 ± 2.8%	Microvascular endothelial cells	In vitro, NIH-3T3 cells; In vivo, SD rats, STZ model	In vitro, 2 mg/mL In vivo, 15 μg/kg	Tail vein 12 weeks,	PI3K\AKT-BCL2	Inhibits apoptosis, increases myocardial capillary density, and improves cardiac function and myocardial ultrastructure	[138]

ROS, reactive oxygen species; BCL-2, B-cell lymphoma/leukemia-2; bFGF, basic fibroblast growth factor; PI3K, phosphatidylinositol 3-kinase; AKT, protein kinase B.

**Table 6:** Active Targeted Delivery in Cardiomyopathy. .

No.	Drug	NPs	Targeting Ligand	Target Organ	Nanoparticle Parameters				Targeted Cells	Study Design	Therapeutic Schedule	Administration	Pathway	Effect	Ref.
					Size	Zeta Potential	PDI	Drug Loading/EE							
1	Argonaute-2	Iron oxide NPs	CHP and SS-1 peptides	Heart/myocardium	57.8 ± 5.7 nm	−1.58 mV at 20 μg	<0.5		Cardiac muscle mitochondria	In vitro, HL-1 cells; In vivo, BKS-db mice	In vitro, 50 μg/mL In vivo, 20 μg; 0.1 mmol/kg body weight	Intravenous 14 days	ROS	Restores mitochondrial homeostasis and alleviates oxidative stress	[140]
2	17β-estradiol	PLGA	PCM-specific peptide	Heart/myocardium	418 ± 11 nm	−20 ± 1 mV	-	84.3 ± 2.8%	Primary cardiomyocyte	In vivo, SD rat	0.4 mg/kg	Tail vein 6 weeks	β-MHC, Col1, Col3	Reduces myocardial cell cross-sectional area and collagen fraction	[141]
3	Baicalin	BA-FFYEEG-ARVYIHPF	AT1R-targeting peptide ARVYIHPF	Heart/myocardium	-	-	-	-	AT1R	In vitro, H9C2 cell In vivo, C57 BL/6 mice	In vivo, 15 mg/kg	Intraperitoneal	SLC7A11-GPX4	Reduces hydrogen peroxide accumulation and inhibits iron overload to improve cardiomyocyte survival	[143]
4	MLN4924	Liposome	Fibroblast membrane-mediated homotypic targeting	Fibrotic myocardium	100–120 nm	−20.2 mV	-	Input ratio: 1:10 (*w*/*w*)	Fibroblasts	In vivo, BALB/c mice	12 mg/kg	Tail vein on days 1–5, 8–12 and 15–20	TGF-β	Inhibits IL-17A and TGF-β signaling, producing anti-inflammatory and anti-fibrotic effects in cardiac fibroblasts	[144]

TGF-β, transforming growth factor beta; IL-17A, interleukin 17A.

## Data Availability

Date are contained within this article.
